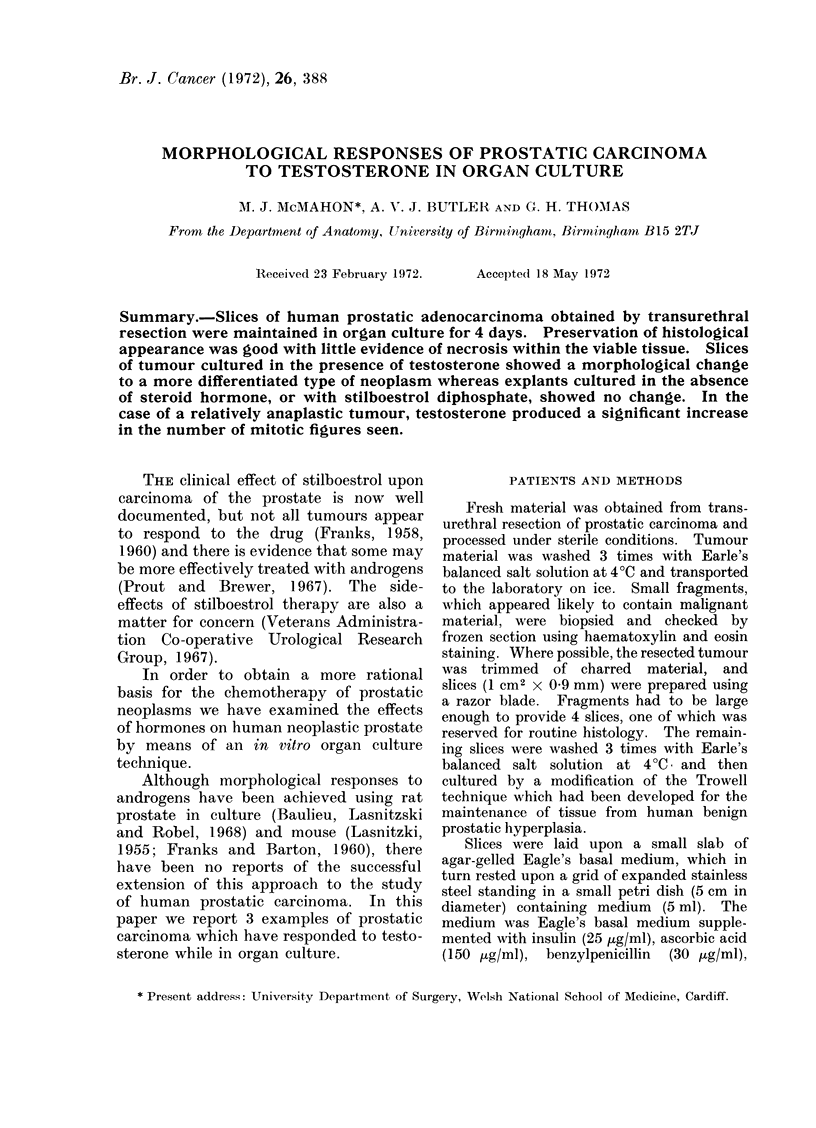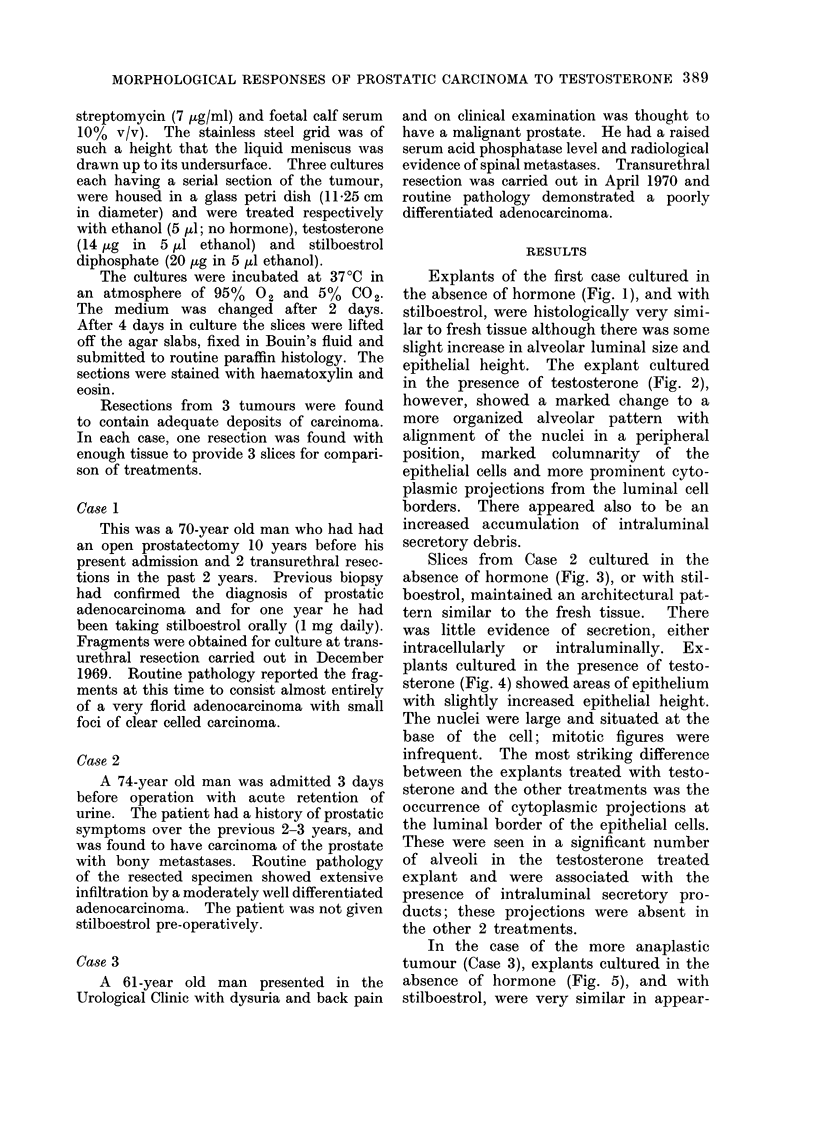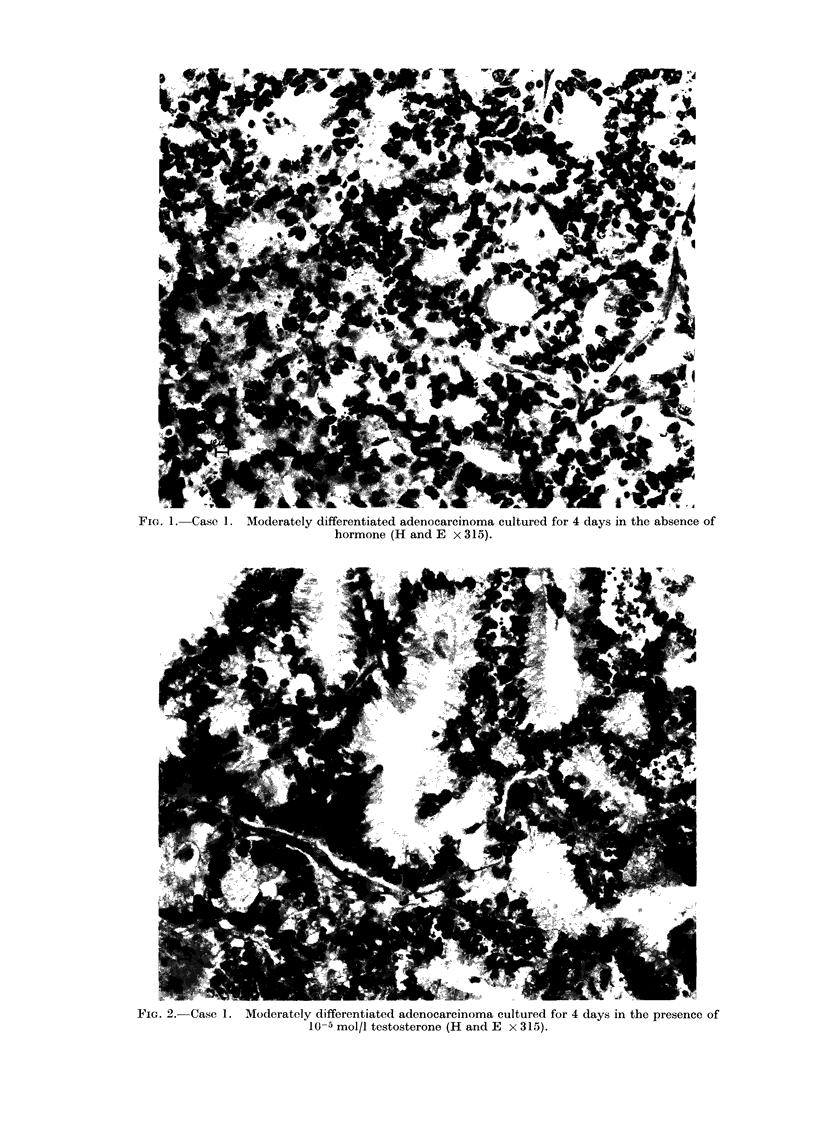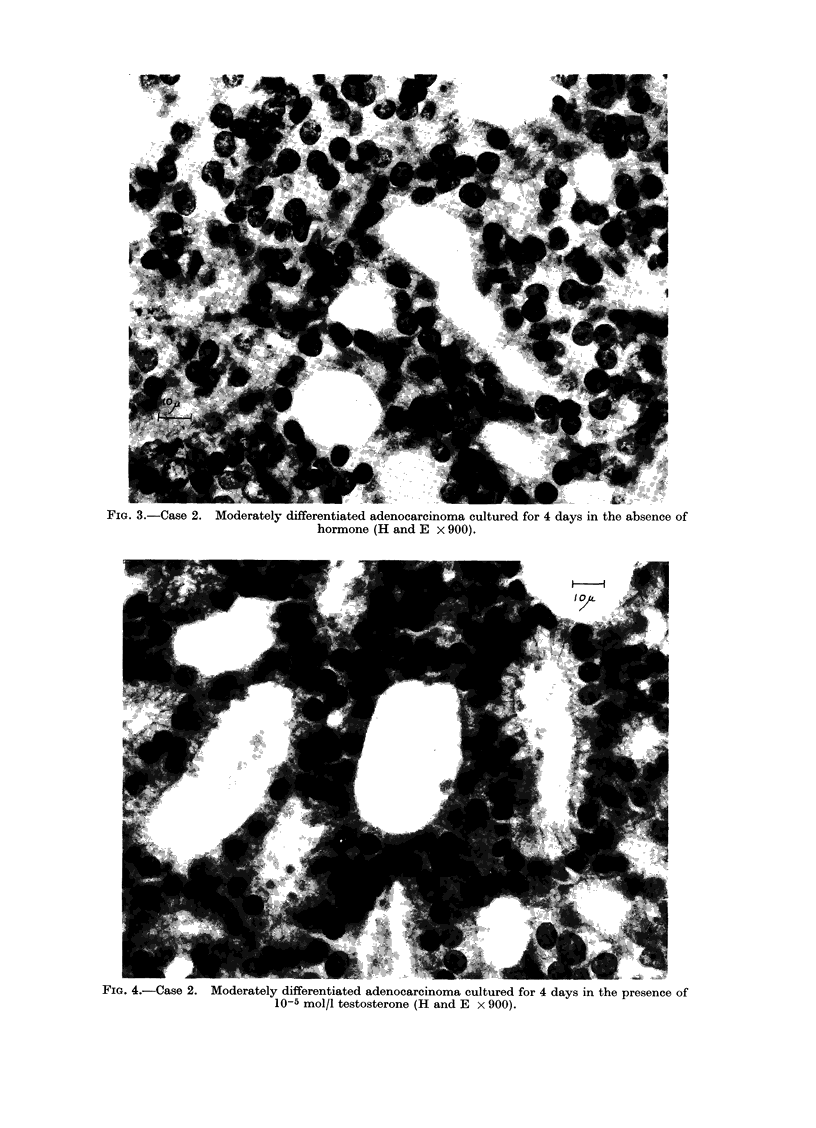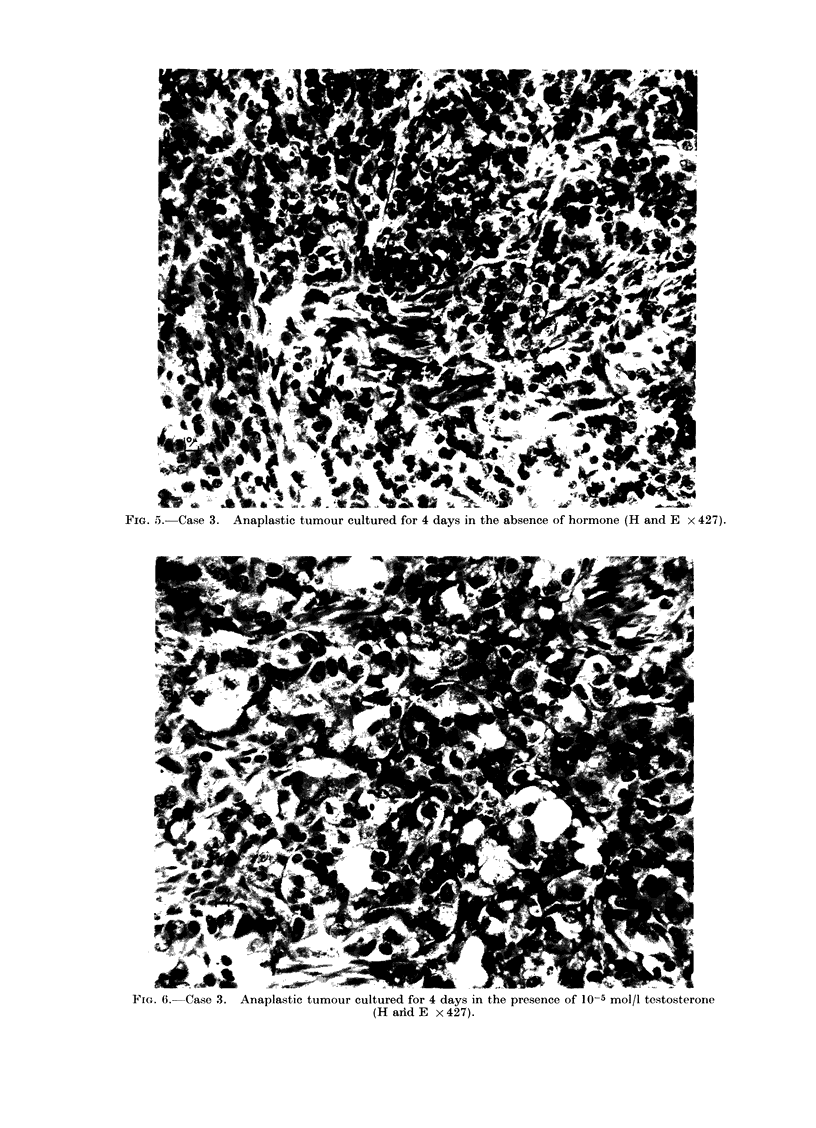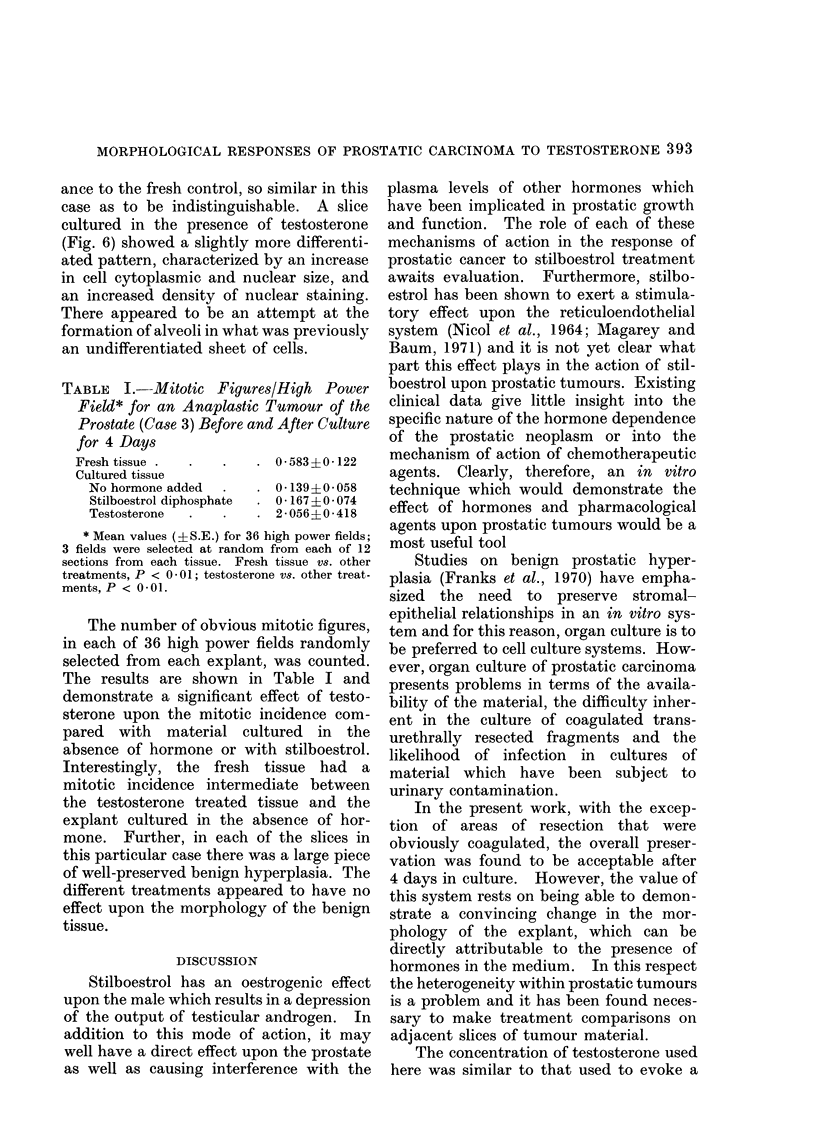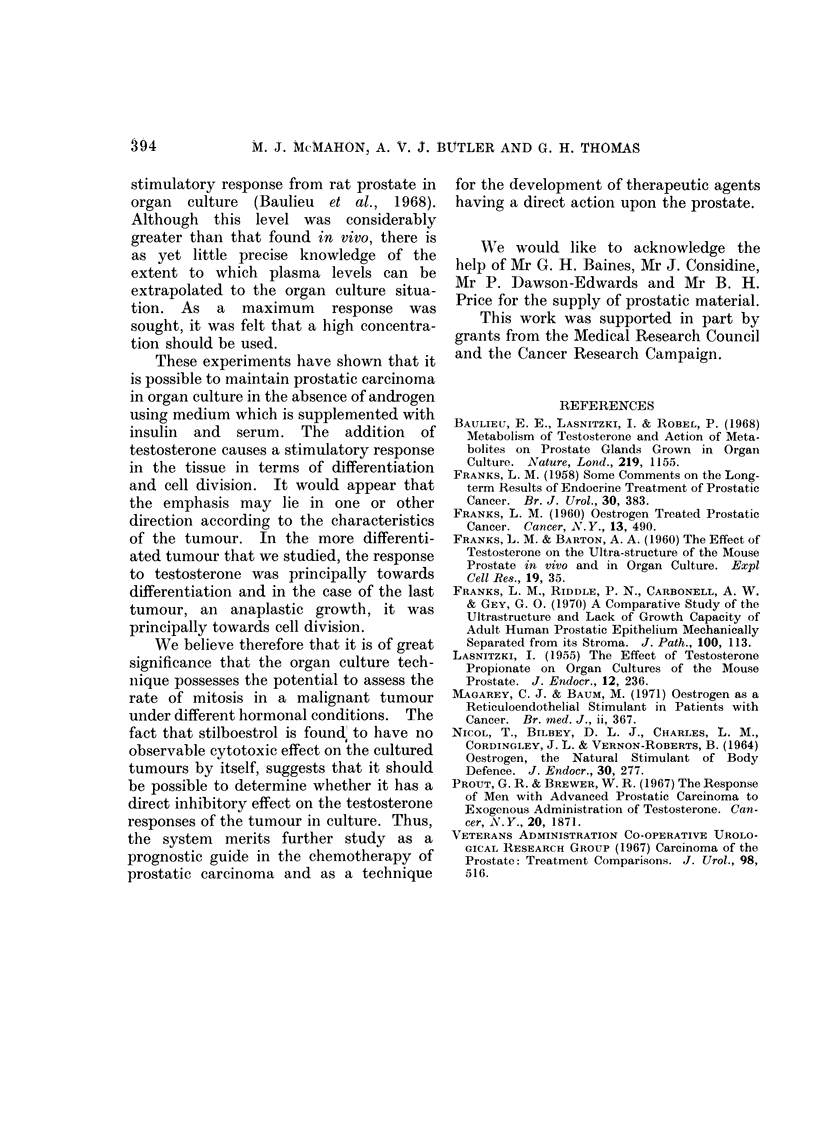# Morphological Responses of Prostatic Carcinoma to Testosterone in Organ Culture

**DOI:** 10.1038/bjc.1972.51

**Published:** 1972-10

**Authors:** M. J. McMahon, A. V. J. Butler, G. H. Thomas

## Abstract

**Images:**


*Br. J. Cancer* (1972), **26**, 388

### MORPHOLOGICAL RESPONSES OF PROSTATIC CARCINOMA TO TESTOSTERONE IN ORGAN CULTURE

M. J. McMAHON*, A. V. J. BUTLER AND G. H. THOMAS

*From the Department of Anatomy, University of Birmingham, Birmingham B15 2TJ*

Received 23 February 1972. Accepted 18 May 1972

**Summary.—Slices of human prostatic adenocarcinoma obtained by transurethral resection were maintained in organ culture for 4 days. Preservation of histological appearance was good with little evidence of necrosis within the viable tissue. Slices of tumour cultured in the presence of testosterone showed a morphological change to a more differentiated type of neoplasm whereas explants cultured in the absence of steroid hormone, or with stilboestrol diphosphate, showed no change. In the case of a relatively anaplastic tumour, testosterone produced a significant increase in the number of mitotic figures seen.**

THE clinical effect of stilboestrol upon carcinoma of the prostate is now well documented, but not all tumours appear to respond to the drug (Franks, 1958, 1960) and there is evidence that some may be more effectively treated with androgens (Prout and Brewer, 1967). The side-effects of stilboestrol therapy are also a matter for concern (Veterans Administration Co-operative Urological Research Group, 1967).

In order to obtain a more rational basis for the chemotherapy of prostatic neoplasms we have examined the effects of hormones on human neoplastic prostate by means of an *in vitro* organ culture technique.

Although morphological responses to androgens have been achieved using rat prostate in culture (Baulieu, Lasnitzski and Robel, 1968) and mouse (Lasnitzki, 1955; Franks and Barton, 1960), there have been no reports of the successful extension of this approach to the study of human prostatic carcinoma. In this paper we report 3 examples of prostatic carcinoma which have responded to testosterone while in organ culture.

### PATIENTS AND METHODS

Fresh material was obtained from transurethral resection of prostatic carcinoma and processed under sterile conditions. Tumour material was washed 3 times with Earle's balanced salt solution at 4 °C and transported to the laboratory on ice. Small fragments, which appeared likely to contain malignant material, were biopsied and checked by frozen section using haematoxylin and eosin staining. Where possible, the resected tumour was trimmed of charred material, and slices (1 cm$^2$ $\times$ 0·9 mm) were prepared using a razor blade. Fragments had to be large enough to provide 4 slices, one of which was reserved for routine histology. The remaining slices were washed 3 times with Earle's balanced salt solution at 4 °C and then cultured by a modification of the Trowell technique which had been developed for the maintenance of tissue from human benign prostatic hyperplasia.

Slices were laid upon a small slab of agar-gelled Eagle's basal medium, which in turn rested upon a grid of expanded stainless steel standing in a small petri dish (5 cm in diameter) containing medium (5 ml). The medium was Eagle's basal medium supplemented with insulin (25 $\mu$g/ml), ascorbic acid (150 $\mu$g/ml), benzylpenicillin (30 $\mu$g/ml),

* Present address: University Department of Surgery, Welsh National School of Medicine, Cardiff.

streptomycin (7 $\mu$g/ml) and foetal calf serum 10% v/v). The stainless steel grid was of such a height that the liquid meniscus was drawn up to its undersurface. Three cultures each having a serial section of the tumour, were housed in a glass petri dish (11·25 cm in diameter) and were treated respectively with ethanol (5 $\mu$l; no hormone), testosterone (14 $\mu$g in 5 $\mu$l ethanol) and stilboestrol diphosphate (20 $\mu$g in 5 $\mu$l ethanol).

The cultures were incubated at 37°C in an atmosphere of 95% $O_2$ and 5% $CO_2$. The medium was changed after 2 days. After 4 days in culture the slices were lifted off the agar slabs, fixed in Bouin's fluid and submitted to routine paraffin histology. The sections were stained with haematoxylin and eosin.

Resections from 3 tumours were found to contain adequate deposits of carcinoma. In each case, one resection was found with enough tissue to provide 3 slices for comparison of treatments.

*Case* 1

This was a 70-year old man who had had an open prostatectomy 10 years before his present admission and 2 transurethral resections in the past 2 years. Previous biopsy had confirmed the diagnosis of prostatic adenocarcinoma and for one year he had been taking stilboestrol orally (1 mg daily). Fragments were obtained for culture at transurethral resection carried out in December 1969. Routine pathology reported the fragments at this time to consist almost entirely of a very florid adenocarcinoma with small foci of clear celled carcinoma.

*Case* 2

A 74-year old man was admitted 3 days before operation with acute retention of urine. The patient had a history of prostatic symptoms over the previous 2–3 years, and was found to have carcinoma of the prostate with bony metastases. Routine pathology of the resected specimen showed extensive infiltration by a moderately well differentiated adenocarcinoma. The patient was not given stilboestrol pre-operatively.

*Case* 3

A 61-year old man presented in the Urological Clinic with dysuria and back pain and on clinical examination was thought to have a malignant prostate. He had a raised serum acid phosphatase level and radiological evidence of spinal metastases. Transurethral resection was carried out in April 1970 and routine pathology demonstrated a poorly differentiated adenocarcinoma.

### RESULTS

Explants of the first case cultured in the absence of hormone (Fig. 1), and with stilboestrol, were histologically very similar to fresh tissue although there was some slight increase in alveolar luminal size and epithelial height. The explant cultured in the presence of testosterone (Fig. 2), however, showed a marked change to a more organized alveolar pattern with alignment of the nuclei in a peripheral position, marked columnarity of the epithelial cells and more prominent cytoplasmic projections from the luminal cell borders. There appeared also to be an increased accumulation of intraluminal secretory debris.

Slices from Case 2 cultured in the absence of hormone (Fig. 3), or with stilboestrol, maintained an architectural pattern similar to the fresh tissue. There was little evidence of secretion, either intracellularly or intraluminally. Explants cultured in the presence of testosterone (Fig. 4) showed areas of epithelium with slightly increased epithelial height. The nuclei were large and situated at the base of the cell; mitotic figures were infrequent. The most striking difference between the explants treated with testosterone and the other treatments was the occurrence of cytoplasmic projections at the luminal border of the epithelial cells. These were seen in a significant number of alveoli in the testosterone treated explant and were associated with the presence of intraluminal secretory products; these projections were absent in the other 2 treatments.

In the case of the more anaplastic tumour (Case 3), explants cultured in the absence of hormone (Fig. 5), and with stilboestrol, were very similar in appear-

FIG. 1.—Case 1. Moderately differentiated adenocarcinoma cultured for 4 days in the absence of hormone (H and E $\times 315$).

FIG. 2.—Case 1. Moderately differentiated adenocarcinoma cultured for 4 days in the presence of $10^{-5}$ mol/l testosterone (H and E $\times 315$).

FIG. 3.—Case 2. Moderately differentiated adenocarcinoma cultured for 4 days in the absence of hormone (H and E ×900).

FIG. 4.—Case 2. Moderately differentiated adenocarcinoma cultured for 4 days in the presence of $10^{-5}$ mol/l testosterone (H and E ×900).

FIG. 5.—Case 3. Anaplastic tumour cultured for 4 days in the absence of hormone (H and E $\times 427$).

FIG. 6.—Case 3. Anaplastic tumour cultured for 4 days in the presence of $10^{-5}$ mol/l testosterone (H and E $\times 427$).

ance to the fresh control, so similar in this case as to be indistinguishable. A slice cultured in the presence of testosterone (Fig. 6) showed a slightly more differentiated pattern, characterized by an increase in cell cytoplasmic and nuclear size, and an increased density of nuclear staining. There appeared to be an attempt at the formation of alveoli in what was previously an undifferentiated sheet of cells.

TABLE I.—*Mitotic Figures/High Power Field* for an Anaplastic Tumour of the Prostate (Case 3) Before and After Culture for 4 Days*

| | |
|---|---|
| Fresh tissue | 0·583±0·122 |
| Cultured tissue | |
| No hormone added | 0·139±0·058 |
| Stilboestrol diphosphate | 0·167±0·074 |
| Testosterone | 2·056±0·418 |

* Mean values (±S.E.) for 36 high power fields; 3 fields were selected at random from each of 12 sections from each tissue. Fresh tissue *vs.* other treatments, $P < 0{\cdot}01$; testosterone *vs.* other treatments, $P < 0{\cdot}01$.

The number of obvious mitotic figures, in each of 36 high power fields randomly selected from each explant, was counted. The results are shown in Table I and demonstrate a significant effect of testosterone upon the mitotic incidence compared with material cultured in the absence of hormone or with stilboestrol. Interestingly, the fresh tissue had a mitotic incidence intermediate between the testosterone treated tissue and the explant cultured in the absence of hormone. Further, in each of the slices in this particular case there was a large piece of well-preserved benign hyperplasia. The different treatments appeared to have no effect upon the morphology of the benign tissue.

### DISCUSSION

Stilboestrol has an oestrogenic effect upon the male which results in a depression of the output of testicular androgen. In addition to this mode of action, it may well have a direct effect upon the prostate as well as causing interference with the plasma levels of other hormones which have been implicated in prostatic growth and function. The role of each of these mechanisms of action in the response of prostatic cancer to stilboestrol treatment awaits evaluation. Furthermore, stilboestrol has been shown to exert a stimulatory effect upon the reticuloendothelial system (Nicol *et al.*, 1964; Magarey and Baum, 1971) and it is not yet clear what part this effect plays in the action of stilboestrol upon prostatic tumours. Existing clinical data give little insight into the specific nature of the hormone dependence of the prostatic neoplasm or into the mechanism of action of chemotherapeutic agents. Clearly, therefore, an *in vitro* technique which would demonstrate the effect of hormones and pharmacological agents upon prostatic tumours would be a most useful tool

Studies on benign prostatic hyperplasia (Franks *et al.*, 1970) have emphasized the need to preserve stromal–epithelial relationships in an *in vitro* system and for this reason, organ culture is to be preferred to cell culture systems. However, organ culture of prostatic carcinoma presents problems in terms of the availability of the material, the difficulty inherent in the culture of coagulated transurethrally resected fragments and the likelihood of infection in cultures of material which have been subject to urinary contamination.

In the present work, with the exception of areas of resection that were obviously coagulated, the overall preservation was found to be acceptable after 4 days in culture. However, the value of this system rests on being able to demonstrate a convincing change in the morphology of the explant, which can be directly attributable to the presence of hormones in the medium. In this respect the heterogeneity within prostatic tumours is a problem and it has been found necessary to make treatment comparisons on adjacent slices of tumour material.

The concentration of testosterone used here was similar to that used to evoke a stimulatory response from rat prostate in organ culture (Baulieu *et al.*, 1968). Although this level was considerably greater than that found *in vivo*, there is as yet little precise knowledge of the extent to which plasma levels can be extrapolated to the organ culture situation. As a maximum response was sought, it was felt that a high concentration should be used.

These experiments have shown that it is possible to maintain prostatic carcinoma in organ culture in the absence of androgen using medium which is supplemented with insulin and serum. The addition of testosterone causes a stimulatory response in the tissue in terms of differentiation and cell division. It would appear that the emphasis may lie in one or other direction according to the characteristics of the tumour. In the more differentiated tumour that we studied, the response to testosterone was principally towards differentiation and in the case of the last tumour, an anaplastic growth, it was principally towards cell division.

We believe therefore that it is of great significance that the organ culture technique possesses the potential to assess the rate of mitosis in a malignant tumour under different hormonal conditions. The fact that stilboestrol is found to have no observable cytotoxic effect on the cultured tumours by itself, suggests that it should be possible to determine whether it has a direct inhibitory effect on the testosterone responses of the tumour in culture. Thus, the system merits further study as a prognostic guide in the chemotherapy of prostatic carcinoma and as a technique for the development of therapeutic agents having a direct action upon the prostate.

We would like to acknowledge the help of Mr G. H. Baines, Mr J. Considine, Mr P. Dawson-Edwards and Mr B. H. Price for the supply of prostatic material.

This work was supported in part by grants from the Medical Research Council and the Cancer Research Campaign.

### REFERENCES

Baulieu, E. E., Lasnitzki, I. & Robel, P. (1968) Metabolism of Testosterone and Action of Metabolites on Prostate Glands Grown in Organ Culture. *Nature, Lond.*, **219**, 1155.

Franks, L. M. (1958) Some Comments on the Long-term Results of Endocrine Treatment of Prostatic Cancer. *Br. J. Urol.*, **30**, 383.

Franks, L. M. (1960) Oestrogen Treated Prostatic Cancer. *Cancer, N.Y.*, **13**, 490.

Franks, L. M. & Barton, A. A. (1960) The Effect of Testosterone on the Ultra-structure of the Mouse Prostate *in vivo* and in Organ Culture. *Expl Cell Res.*, **19**, 35.

Franks, L. M., Riddle, P. N., Carbonell, A. W. & Gey, G. O. (1970) A Comparative Study of the Ultrastructure and Lack of Growth Capacity of Adult Human Prostatic Epithelium Mechanically Separated from its Stroma. *J. Path.*, **100**, 113.

Lasnitzki, I. (1955) The Effect of Testosterone Propionate on Organ Cultures of the Mouse Prostate. *J. Endocr.*, **12**, 236.

Magarey, C. J. & Baum, M. (1971) Oestrogen as a Reticuloendothelial Stimulant in Patients with Cancer. *Br. med. J.*, ii, 367.

Nicol, T., Bilbey, D. L. J., Charles, L. M., Cordingley, J. L. & Vernon-Roberts, B. (1964) Oestrogen, the Natural Stimulant of Body Defence. *J. Endocr.*, **30**, 277.

Prout, G. R. & Brewer, W. R. (1967) The Response of Men with Advanced Prostatic Carcinoma to Exogenous Administration of Testosterone. *Cancer, N.Y.*, **20**, 1871.

Veterans Administration Co-operative Urological Research Group (1967) Carcinoma of the Prostate: Treatment Comparisons. *J. Urol.*, **98**, 516.